# Swenson-like pull-through for treatment of the rare association between Hirschsprung’s disease and anorectal malformation

**DOI:** 10.1186/s12887-025-05549-0

**Published:** 2025-03-25

**Authors:** Mohamed Abdelmalak, Mohamed Mansy, Hazem Khafaga, Yasmine Ghazaly, Reem Saeed, Nada Yakout, Saber Waheeb, Mostafa Zain

**Affiliations:** 1 Nile of Hope Hospital for Congenital Anomalies, Alexandria, Egypt; 2 Faculty of Medicine, Port Said University, Port Said, Egypt; 3 Faculty of Medicine, Alexandria University, Alexandria, Egypt

**Keywords:** Swenson-like Pull-through, Hirschsprung’s Disease, Anorectal Malformation, Colorectal surgery

## Abstract

**Background:**

Anorectal malformations and Hirschsprung’s disease are congenital conditions impacting the digestive system, with a particularly uncommon co-occurrence, estimated at 2–3% of all ARM cases. This case series explores this rare association through three distinct cases, each presenting unique clinical challenges and insights.

**Methods:**

We report a series of five patients with ARM who were concurrently diagnosed with HD based on clinical and radiological evaluations, with definitive confirmation obtained through rectal biopsy. In cases where HD was diagnosed after the complete surgical repair of ARM, the patients underwent a Swenson-like pull-through procedure. Notably, the anastomosis was created approximately 3 cm from the anal verge, rather than the conventional 3 cm from the dentate line.

**Results:**

This study reviewed the records of 136 ARM patients treated at our center over five years, identifying five cases with concurrent HD. In three of these cases, HD was initially overlooked and diagnosed only after ARM repair. These patients underwent a Swenson-like pull-through procedure. During follow-up, two patients achieved good bowel control without fecal soiling, while the third had regular bowel movements and satisfactory growth, albeit with occasional episodes of enterocolitis managed medically. The remaining two cases were identified earlier, following colostomy, which led to a different management approach.

**Conclusion:**

This case series underscores the critical importance of considering HD in patients with ARM who present with persistent, atypical gastrointestinal symptoms post-surgical repair of their ARM. Preservation of the aganglionic neoanal canal with a subsequent Swenson pull-through appeared beneficial to achieve good postoperative continence.

## Introduction

Anorectal malformation (ARM) and Hirschsprung disease (HD) are two distinct but significant congenital conditions affecting the pediatric population each with a prevalence of 1: 4000–5000 live births [1, 2]. ARM is a developmental structural anomaly of the anus and rectum ranging from low anomalies to more severe forms like cloaca and high anomalies [3]. On the other hand, Hirschsprung disease is a condition impacting the development of the enteric nervous system, particularly the nerve cells in the distal colon and rectum. This lack of nerve cells hinders proper intestinal muscle contraction, leading to symptoms like constipation and bloating due to stool blockage [4, 5].

The coexistence of ARM and HD is a rare phenomenon, estimated to occur in about 2–3% of ARM cases, and was first reported in the literature by Kiesewetter et al. in 1964 [6]. This unusual association is not only intriguing but also clinically significant. Understanding the simultaneous occurrence of these conditions is vital due to the diagnostic and therapeutic challenges it poses [7]. The literature about this association primarily consists of single case reports and limited case series [8–11]. These studies indicate a growing interest in this association but also highlight the need for more comprehensive research [12].The etiology of this association is still obscured and unknown. In the reported cases, there were many suggested explanations including genetic, familial, and environmental factors however, there were no single factor or groups of factors noted to be responsible for the simultaneous association of ARM and HD. According to Puri et al., the explanation of the pathophysiological basis of this association was poorly understood [13].

In this article, we present our institution's experience with this rare association, highlighting the warning signs that can facilitate early diagnosis to prevent unnecessary surgical interventions and reduce morbidity. Additionally, we describe a Swenson-like pull-through technique that was performed in cases where the diagnosis of HD was established after the complete surgical repair of the ARM.

## Methods

This study reviewed the medical records of patients with ARM who were concurrently diagnosed with HD at our institution over 5 years (from 2020 to 2024). Diagnosis of concomitant HD was based primarily on clinical and radiological evaluations, with definitive confirmation obtained through rectal biopsy. In cases where HD was diagnosed after the complete surgical repair of the ARM, a Swenson-like pull-through procedure was performed.

### Description of the technique

Initiated with an abdominal approach, the operation involves a meticulous devascularization of the segment intended for resection. Care should be taken to preserve a margin of approximately 3–4 cm proximal to the anocutaneous junction, ensuring an adequate blood supply for the remaining tissue. The next step is carefully mobilizing a healthy, normally functioning ganglionic colonic segment. This step is crucial to facilitate the subsequent pull-through of the colon to the anorectum. Intraoperative frozen section histopathology is used to confirm the presence of ganglion cells at the proximal resection margin. Once the colon is fully mobilized, it is delicately brought out through the newly created anal opening, ensuring proper alignment and orientation for the anastomosis. The colo-colonic anastomosis is then constructed externally at the anal verge. Upon completion of the anastomosis, the colon is gently reduced back into the pelvic cavity. A rectal tube is inserted to ensure patency and support healing. Depending on the individual case, a protective colostomy may be considered to safeguard the anastomosis and facilitate recovery.

Data were collected on patient demographics, types of ARM, associated anomalies, presenting symptoms, diagnostic investigations, details of pull-through surgeries, and postoperative follow-up. Ethical approval was obtained from the institutional ethics committee, and all patient data were anonymized to ensure confidentiality.

## Results

This retrospective study analyzed the medical records of patients with ARM at our institution over a five-year period (2020–2024). During this timeframe, a total of 136 patients were identified as having ARM. Among these, five cases were found to have a concurrent diagnosis of HD. In three of these cases, HD was not initially recognized and was only diagnosed after the patients had undergone complete surgical repair of ARM. Following the confirmation of HD, these patients subsequently underwent a Swenson-like pull-through procedure to address the condition. In contrast, the remaining two cases were diagnosed with HD at an earlier stage, following the creation of a colostomy.

### Case 1

A 5-year-old male patient presented to our center complaining of severe constipation, with bowel movements occurring only once every ten days, accompanied by recurrent abdominal pain and distension. The patient has a history of ARM (recto-prostatic fistula) and underwent a three-stage repair during his first year of life. According to the mother, the patient showed no improvement in bowel habits or symptom relief (severe constipation and abdominal distension) despite adhering to a strict laxative diet and receiving maximum doses of laxative medications.

**Fig. 1:**
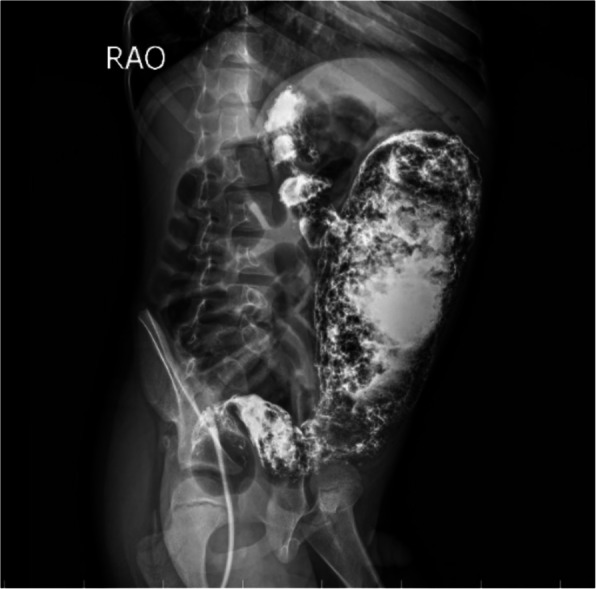
Barium enema shows discrepancy at the level of the sigmoid colon

**Fig. 2:**
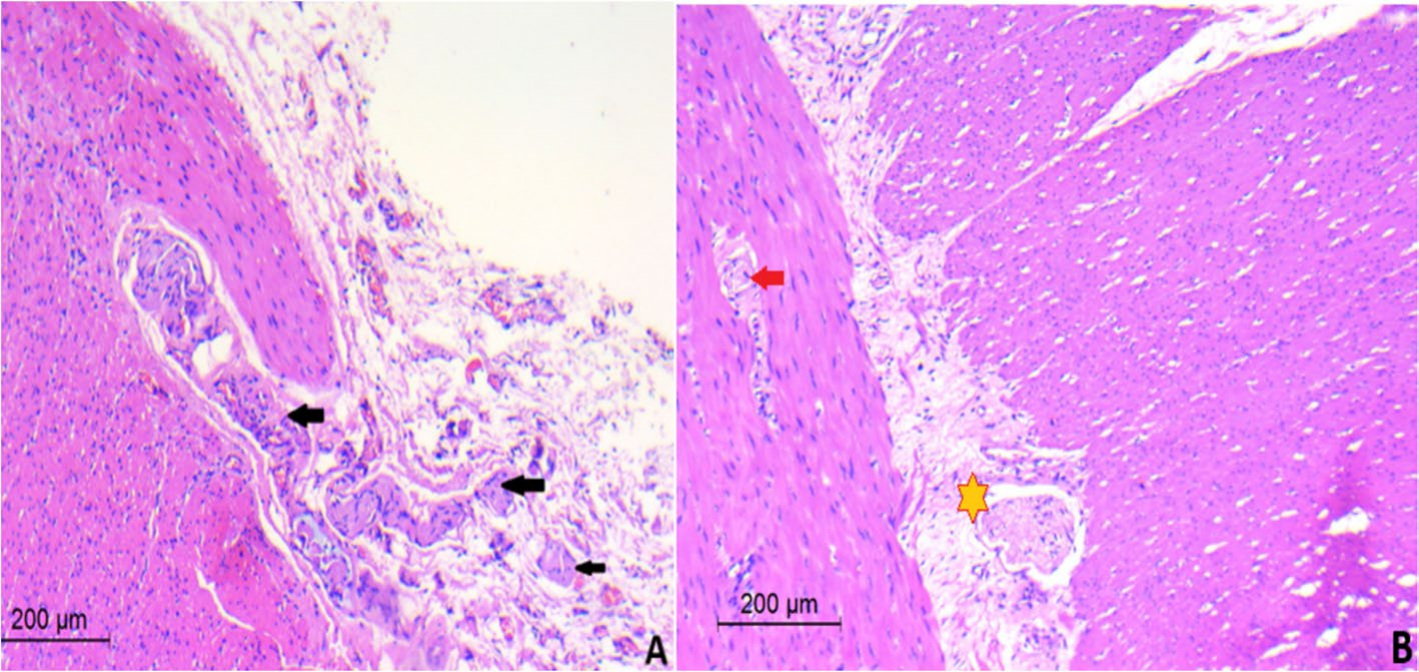
**A** Photomicrograph of submucosa and muscularis propria showing hypertrophic nerves “ Black arrows”. **B** Photomicrograph of submucosa and muscularis propria showing absence of ganglion cells “ Red arrow” and fibromuscular dysplasia of intermuscular blood vessels “ yellow Asterix”

Upon rectal examination, there were no strictures or stenosis. A plain abdominal x-ray showed massive colonic dilatation with a ground glass appearance. To investigate further, a contrast enema was performed, which revealed a discrepancy suggestive of an underlying condition beyond ARM (Fig. 1). A rectal biopsy was then conducted to confirm the suspected diagnosis of HD which demonstrated that the submucosa and musculosa showed the absence of ganglion cells with presence of hypertrophied nerves. (Fig. 2).

**Fig. 3:**
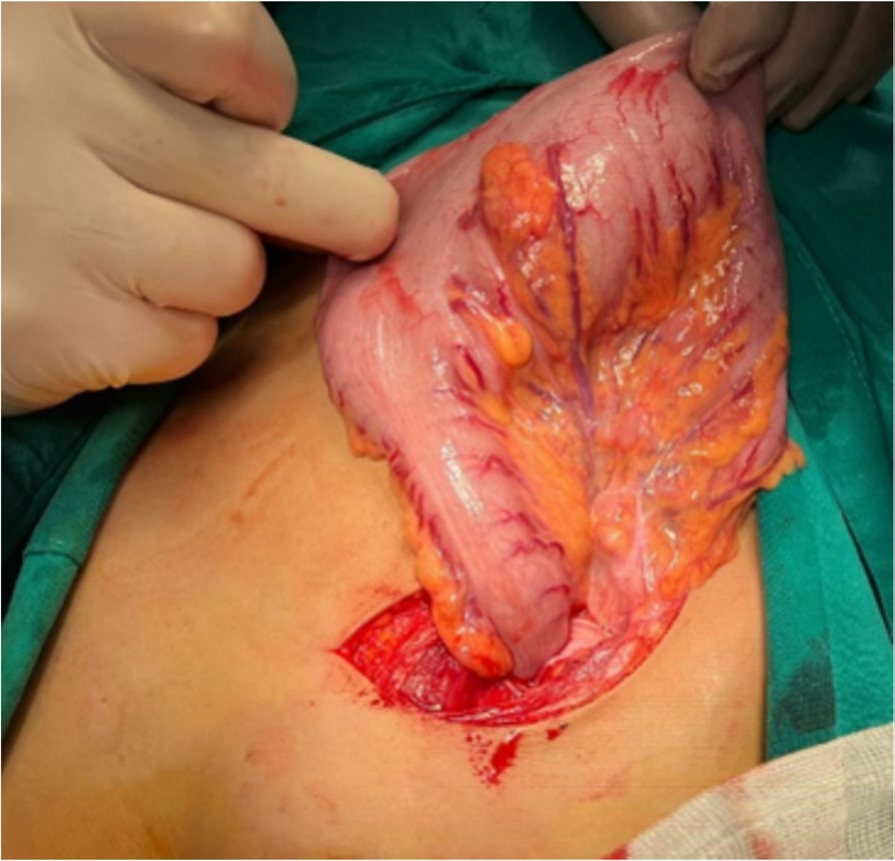
Intraoperative image showing the discrepancy at the level of the sigmoid colon

The diagnosis of HD was confirmed, and the decision was made to perform a pull-through operation. Upon exploration, there was an evident classic discrepancy of HD at the level of the sigmoid colon (Fig. 3). The patient underwent a Swenson-like pull-through procedure where a colonic resection was performed, and the proximal normal caliber bowel was used for the coloanal anastomosis, preserving the previously created anal canal. Aganglionosis extended 8 cm from the rectosigmoid junction. The patient had an uneventful recovery and started oral feeding on the 4th postoperative day. The patient was discharged from the hospital on the 7th postoperative day. The patient experienced a passage of frequent loose motions for the first 2 weeks after the operation which improved gradually.

Two years after the procedure, the patient has shown significant improvement, particularly in achieving spontaneous, regular bowel movements in moderate amounts without the need for laxatives. Following the implementation of a bowel management program, which entailed regular toilet training and adherence to a nutrition protocol incorporating a laxative diet without medications or laxative drugs, the patient now passes stool 1–2 times daily. His continence is well preserved, with good anal sensations and no episodes of stool soiling. The patient had regular bowel habits and no significant limitations in daily activities related to bowel function.

### Case 2

**Fig. 4:**
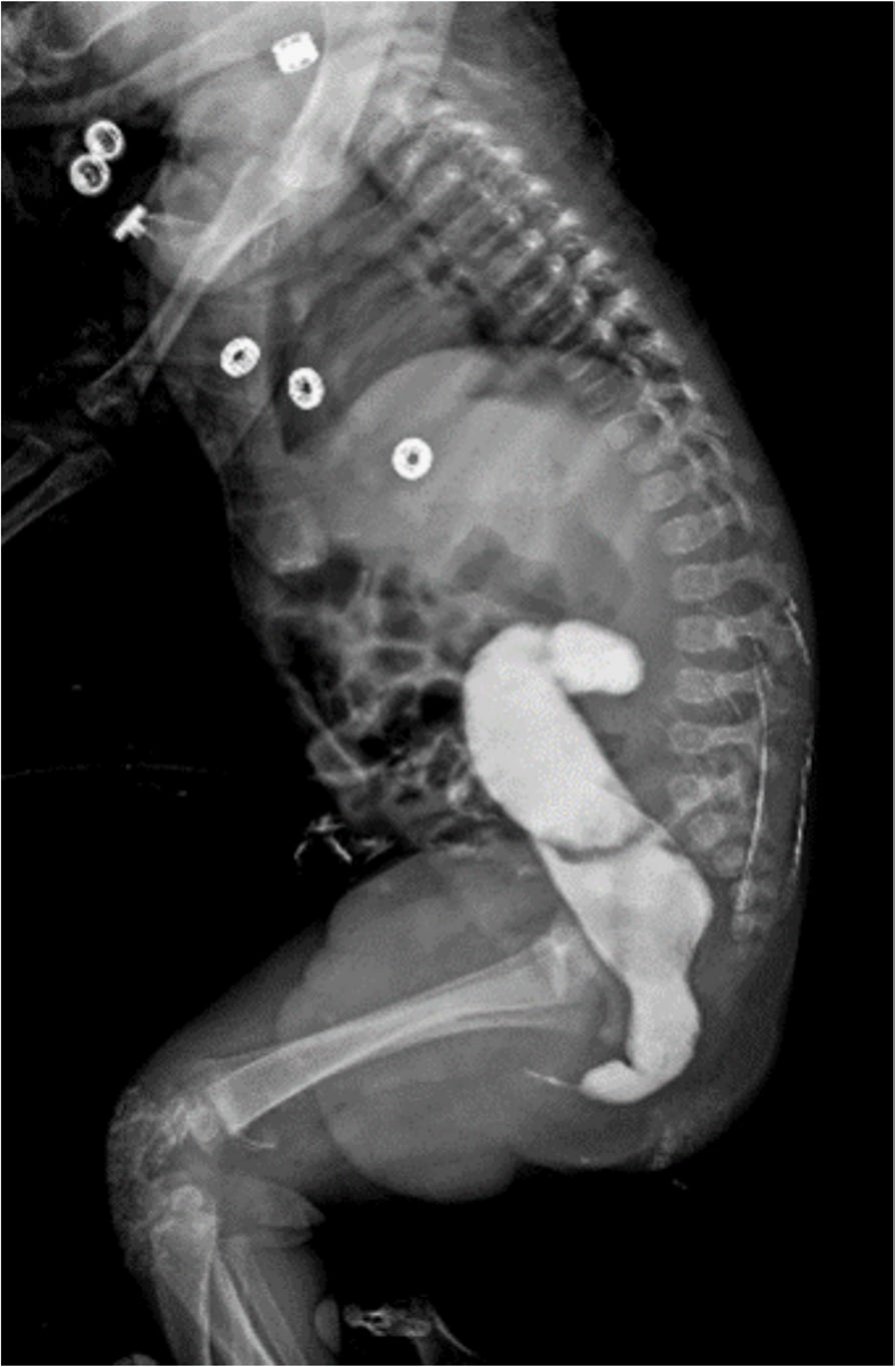
Distal loop colostogram shows the presence of a recto-bulbar fistula with a discrepancy in the colonic caliber at the rectosigmoid level

A 2-month-old male baby with ARM presented to our center with a loop right transverse colostomy, The baby had mild pulmonary artery stenosis, a small atrial septal defect (ASD), and a solitary right kidney. A distal loop colostogram was performed at the age of four months revealing the presence of a recto-bulbar fistula. Also, it showed a discrepancy in the colonic caliber at the rectosigmoid level, but this finding was underestimated at this time and was explained as an artifact or angulation (Fig. 4).

**Fig. 5:**
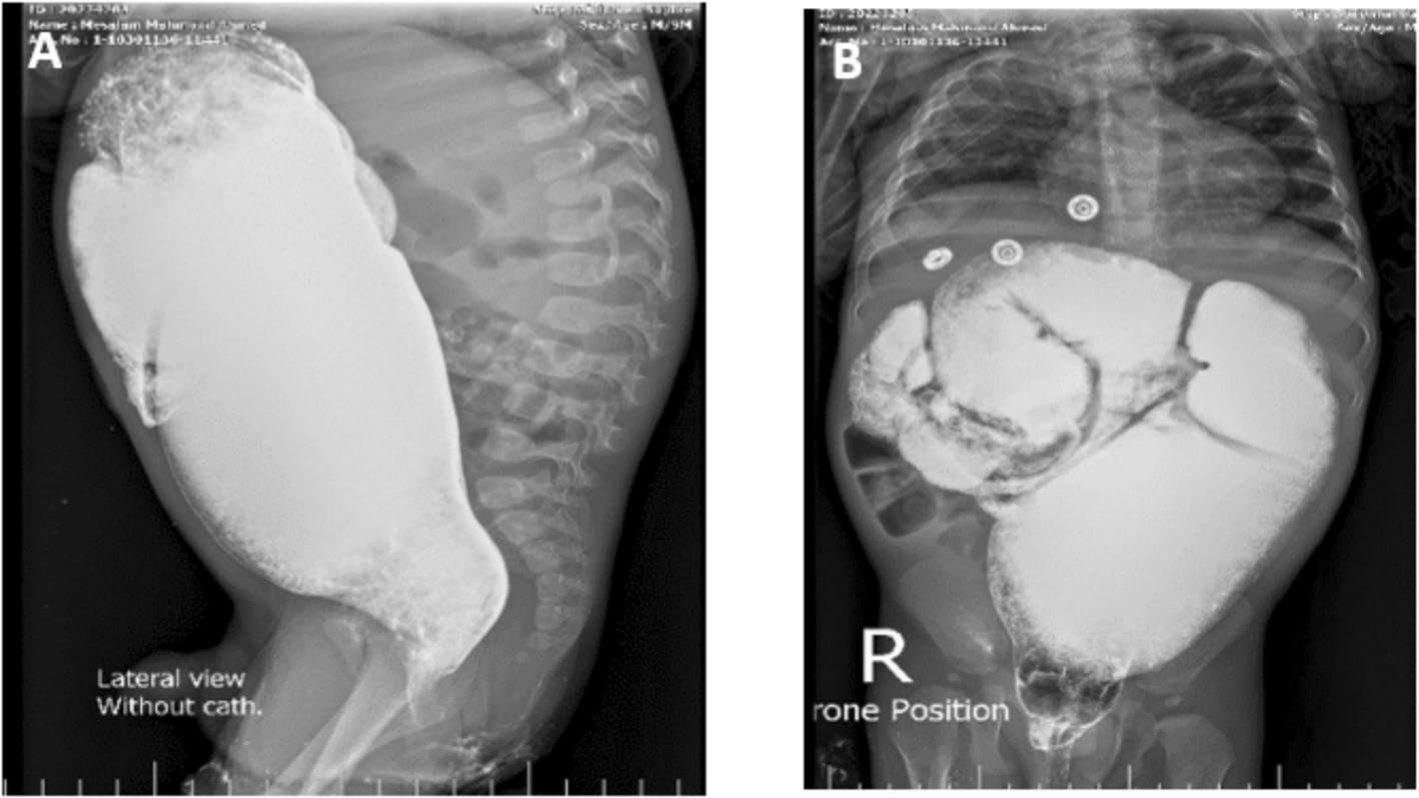
Contrast enema **A** Lateral view film. **B** Anteroposterior view film. Both show a hugely dilated colon with rectosigmoid discrepancy and delayed evacuation

The patient underwent a posterior sagittal anorectoplasty (PSARP) and colostomy closure at the age of 5- and 7-month respectively. After the stoma was closed he experienced obstructive symptoms after the surgery, including the inability to pass stool, abdominal distension, feeding refusal, and recurrent vomiting. The patient was followed in the clinic and treated with oral laxatives and suppositories. After 6 weeks, a contrast enema was done and revealed a rectosigmoid discrepancy with delayed evacuation raising the suspicions for HD (Fig. 5).

**Fig. 6:**
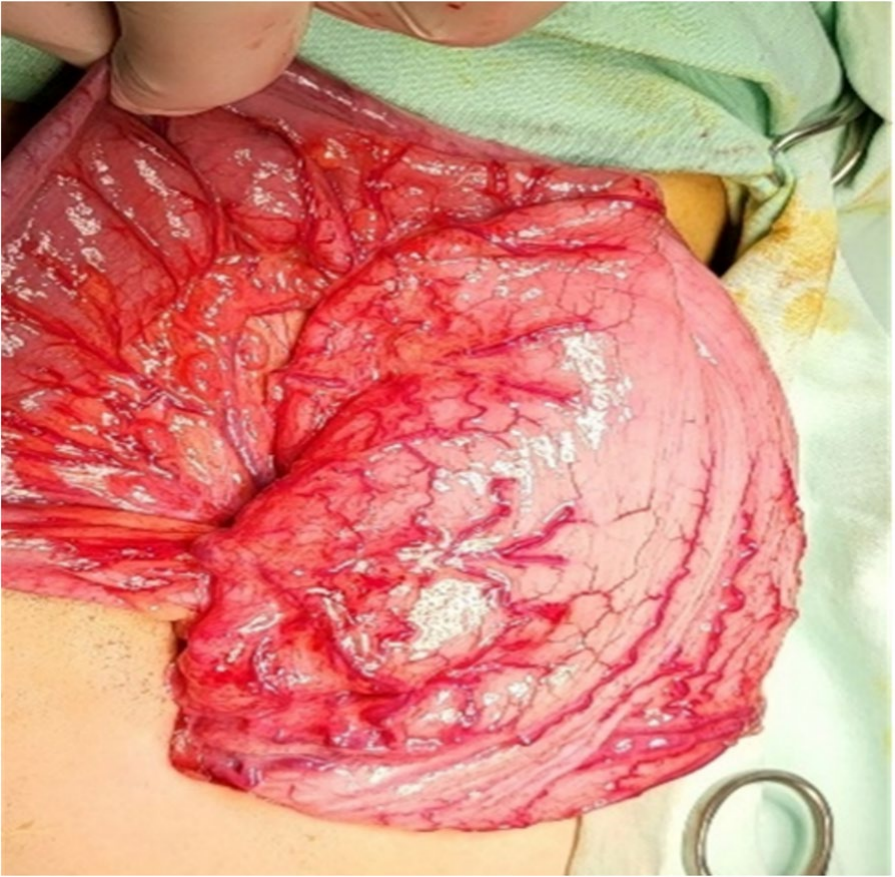
Intraoperative image showing massive dilatation of the sigmoid and descending colon with corkscrew colonic vasa recta

Two months after the operation, the baby presented with severe enterocolitis. Examination revealed high-grade fever, severe abdominal distention, and passage of foul-smelling watery stool. The patient was admitted to the pediatric intensive care unit (PICU) for resuscitation. Despite the maximal supportive measures with intravenous fluids, antibiotics, and metronidazole in addition to colonic wash, the condition of the patient didn’t improve for 48 h. So, the decision was made for surgical exploration. This revealed a markedly dilated sigmoid, descending, and transverse colon with corkscrew colonic vasa recta and a narrow rectum (Fig. 6). A lower descending colostomy was done with full-thickness biopsies from the stoma site, sigmoid colon, and rectum. The patient had a smooth postoperative course. He started oral feeding on the 2nd postoperative day and was discharged on the 5th.

**Fig. 7:**
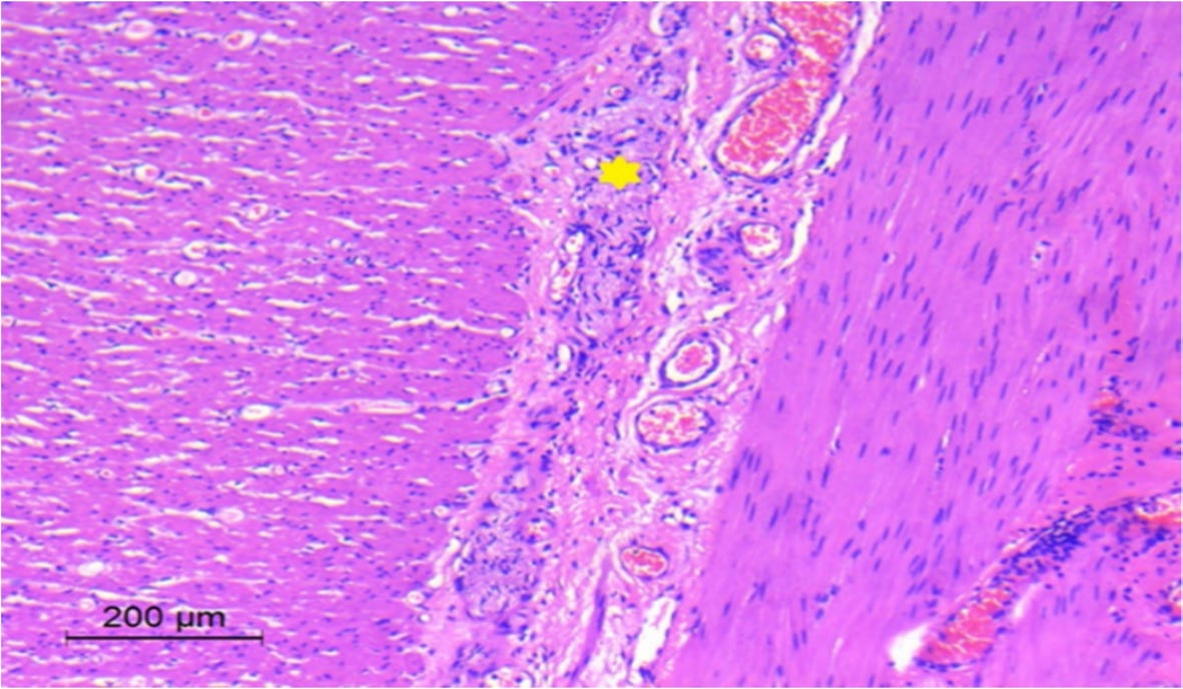
Photomicrograph of submucosa and muscularis propria of the rectum showing hypertrophic nerves “ yellow Asterix”

Histopathological examination of biopsies from the sigmoid colon and rectum showed hypertrophied nerves with no ganglion cells in the submucosa and musculosa propria. The biopsy from the stoma site showed no significant neuromuscular abnormality with normal ganglionic margins (Fig. 7). After 3 months the patient underwent colostomy pull-through using the Modified Swenson-like technique. Aganglionosis involved the rectum and distal 6 cm of the sigmoid colon. The postoperative course for the patient was notably smooth without any complications. He started oral intake on the 6th day after the surgery and was discharged on the 8th. Subsequent follow-up visits showed promising outcomes. The patient established regular bowel motions, passing stool 2–3 times daily. The consistency of the stool was soft, and the color varied from green to yellow within normal expectations for a postoperative patient. After two years of follow-up, the patient maintains regular bowel habits, with 1–3 soft stools passed daily. He has achieved normal weight gain, reflecting an overall improvement in nutritional status and growth. The mother reports infrequent episodes of enterocolitis, all of which were successfully managed with oral medical treatment. Despite these occasional episodes, the patient experiences no significant disruptions in daily activities, and the bowel management program remains effective in controlling symptoms.

### Case 3

**Fig. 8:**
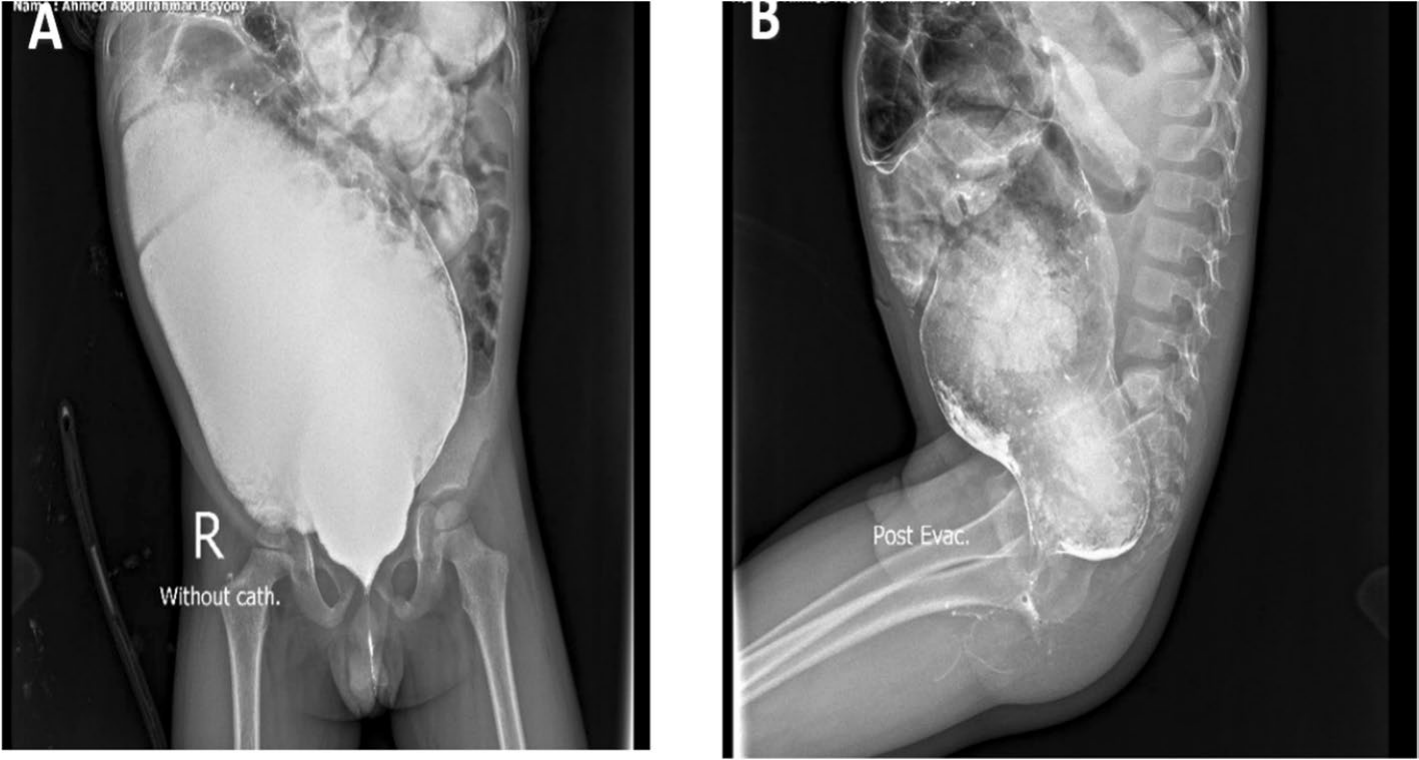
Contrast enema **A** Anteroposterior view film. **B** Lateral view film. Both show a discrepancy at the rectosigmoid area with massive dilatation of the sigmoid colon

**Fig. 9:**
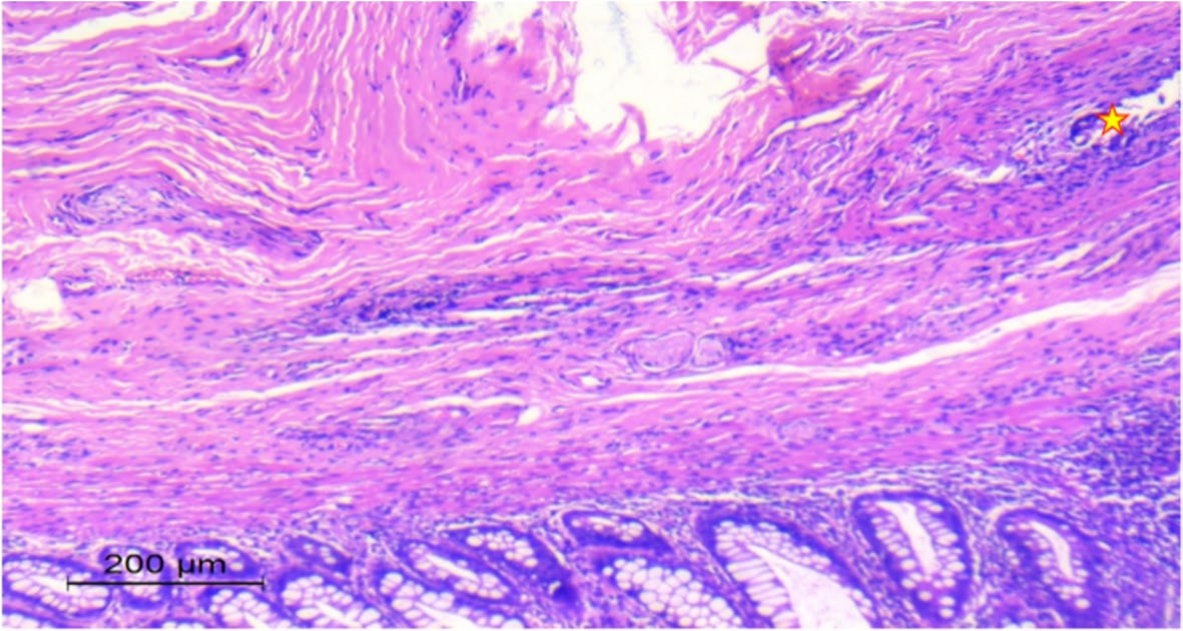
**A** Photomicrograph of submucosa and muscularis propria showing hypertrophic nerves “yellow Asterix”

A 2-year-old male presented to our clinic with a long history of constipation. He has a history of ARM (recto-perineal fistula). He underwent perineal anoplasty on the first postnatal day. The mother that the boy had severe constipation passing stool once every 3–4 days and didn’t respond well to oral laxatives or suppositories. Examination revealed a dilatable anal opening with no stenosis or stricture. The patient was set on a bowel management program and the dose of laxatives was adjusted. The patient was followed for 6 months but did not show any improvement in the ability to pass stool. Given these persistent symptoms, a contrast enema was conducted, which raised suspicions of Hirschsprung disease due to the observed discrepancy at the rectosigmoid area with massive dilatation of the sigmoid colon (Fig. 8). A rectal biopsy demonstrated hypertrophied nerves with absence of ganglion cells in the submucosa and musculosa (Fig. 9).

As the diagnosis of HD was confirmed, an abdominal Swenson like pull-through procedure was performed. The patient exhibits a stable and satisfactory post-operative recovery. He started oral feeding on the 4th postoperative day. The patient was discharged from the hospital on the 7th postoperative day. Histopathological examination of the resected segment confirmed the diagnosis with findings that the submucosa and muscularis showed an absence of ganglion cells with hypertrophied nerves. The proximal resection margin showed rarefaction of muscularis propria. Aganglionosis was limited to the rectum, extending approximately 5 cm proximally.

At the one-year follow-up, the patient has normal stool frequency and characteristics, with 1–2 bowel movements per day. Importantly, the patient does not require laxatives to maintain these regular bowel habits. His continence is excellent, with no reported episodes of fecal soiling. The absence of bowel-related complications has enabled the patient to participate in regular activities without gastrointestinal issues affecting daily life (Table 1).

**Table 1:** Characteristics of the cases

Case	Age	ARM Type	Post-Operative (ARM) Symptoms	Diagnostic approach	Treatment	Outcome
1	4 years	Recto-prostatic	• Persistent constipation • Abdominal distension	• Contrast enema • Rectal biopsy	• Swenson-like pull-through procedure	Resolution of symptoms with a bowel management program
2	1 year	Recto-bulbar	• Severe abdominal distension • Constipation • Enterocolitis	• Contrast enema • Exploration with multiple biopsies	• Colostomy • Swenson-like pull-through procedure	Improvement in symptoms with infrequent attacks of enterocolitis which respond to medical treatment
3	2 years	Rectoperineal	• Persistent constipation	• Contrast enema • Rectal biopsy	• Swenson-like pull-through procedure	Resolution of symptoms without the need for laxatives

### Case 4

A full-term male infant was diagnosed with an ARM (recto-urethral fistula) at birth. A double-barrel descending colostomy was performed on the second day of life. Postoperatively, the infant experienced three episodes of necrotizing enterocolitis, all of which were managed conservatively. At 7 months of age, the infant presented with severe enterocolitis and sepsis. Following stabilization, histopathological biopsies from the stoma and rectum confirmed the diagnosis of HD, with acetylcholinesterase staining providing further diagnostic confirmation. During abdominal exploration, a significant caliber discrepancy in the transverse colon was identified, prompting the decision to perform a leveling colostomy. Three months later, the patient underwent a pull-through procedure, creating a neoanus at the level of the ganglionic transverse colon, as confirmed by intraoperative frozen section histopathology. The rectal fistula was at the level of the bulbar urethra. The postoperative recovery was uneventful.

The child was referred to our center at eight years of age due to persistent fecal incontinence with frequent soiling episodes. A lumbosacral spine MRI was performed to evaluate the presence of associated spinal anomalies. The imaging revealed an absent coccyx and the absence of two sacral segments. Despite extensive bowel management and training efforts, the incontinence persisted. At ten years of age, the patient underwent a Malone antegrade continence enema procedure to address the incontinence. Two years postoperatively, the patient achieved continence with the use of antegrade enemas, demonstrating good bowel function control.

### Case 5

A full-term male infant with Down syndrome was diagnosed at birth with a high ARM without a fistula and Tetralogy of Fallot. On the third day of life, a simple loop right transverse colostomy was performed. During the first three months of life, the infant was hospitalized three times for partial intestinal obstruction, which was managed conservatively with bowel rest, decompression, and intravenous antibiotics.

At four months of age, the infant was referred to our center due to severe failure to thrive, recurrent episodes of enterocolitis, and clinical signs of intestinal dysmotility. Histopathological biopsies obtained from the stoma and rectum confirmed the diagnosis of HD. The decision was made to perform an exploratory laparotomy with colonic mapping and a leveling ileostomy. Histopathological examination with acetylcholinesterase staining revealed total colonic aganglionosis with ileal involvement extending approximately 10 cm proximal to the ileocecal valve. The infant experienced a prolonged postoperative recovery and was discharged after three weeks. However, at six months of age, the infant was readmitted with systemic pseudomonas sepsis and multiorgan failure. Despite intensive medical interventions, including broad-spectrum antibiotics and comprehensive critical care support, the infant unfortunately passed away two weeks later.

## Discussion

The coexistence of HD and ARM poses significant diagnostic and therapeutic challenges. Each case required a tailored approach, reflecting the variability in symptoms, age of presentation, and severity. In a systematic review of reported 42 single cases, 23 cases (54.7%) have been associated with other syndromes such as Down syndrome, Cat eye syndrome, Currarino triad, and Pallister–Hall syndrome. This high incidence of associated syndromes indicates a syndromic pathology of this association; however, it may be partly explained by the local genetic pool of the patients studied. This may affect the future diagnostic strategies for such clinical associations [14, 15]. In this review, two cases (1 and 3) showed no apparent syndromic association. In contrast, Case 2 had mild pulmonary artery stenosis, a small ASD, and a solitary right kidney, while Case 5 was diagnosed with Down syndrome and Tetralogy of Fallot. Also, Case 4 exhibited an absent coccyx and the absence of two sacral segments, findings that may contribute to bowel dysfunction, necessitating an antegrade Malone washout procedure to assist with bowel management.

Identification of this association is usually delayed after full correction of ARM as most cases with high ARM are treated primarily with a diverting colostomy at the neonatal period. Moreover, postoperative symptoms like constipation and abdominal distension can be mistakenly attributed solely to ARM, delaying the consideration of HD [9, 11].

Given the rarity of this association and the overlap in postoperative symptoms between ARM and isolated HD, a multidisciplinary approach involving pediatric surgeons, radiologists, and pathologists is crucial. Early recognition through contrast studies and timely biopsies in patients with atypical postoperative courses, such as persistent constipation or enterocolitis, may facilitate earlier diagnosis and intervention, improving long-term outcomes.

The histopathological examination of our cases revealed classic findings of Hirschsprung Disease, including aganglionosis and hypertrophic nerve endings, particularly in the submucosal and myenteric plexuses. These features are diagnostic of primary aganglionosis, reflecting the congenital etiology of the disease. The identification of hypertrophic nerve fibers underscores the compensatory response to the absence of ganglion cells and guided our surgical strategy to include only ganglionic bowel in the pull-through procedures. The absence of any findings suggestive of secondary aganglionosis further supports a primary, developmental origin in these cases.

The presented case series illustrates the intricate and varied clinical presentations of three unique cases with concurrent ARM and HD. Case 2, involving an infant, highlights the severity and immediacy of postoperative obstructive symptoms, leading to an early diagnosis and intervention. Contrastingly, Case 1, with a 5-year-old patient, demonstrates a more protracted course, with chronic symptoms of constipation and abdominal pain causing a delayed diagnosis. Case 3, involving a toddler, falls between these two extremes, presenting with persistent symptoms despite initial corrective surgery. These varying timelines and symptom severities reflect the diagnostic challenges in identifying the coexistence of ARM and HD, emphasizing the need for a high index of suspicion in patients with intractable constipation after correction of ARM. In cases 4 and 5, the patients presented with recurrent episodes of necrotizing enterocolitis and failure to thrive.

In the series reported by Watanatittan et al. [8], they concluded that the diagnosis was delayed after full correction of ARM due to the atypical symptomatology and radiological findings. According to them, some patients of ARM with associated aganglionosis may not present with the typical symptoms of HD after surgical correction of ARM. Stool soiling or incontinence is a common complaint after the treatment of the intermediate or high type of ARM. If the soiling or incontinence is severe, the symptoms of constipation may not be so conspicuous to the parents. Also, they stated that postoperative constipation may be taken for granted as a complication of surgical treatment of ARM. Indeed, constipation after surgery for ARM is most commonly due to a stricture at the mucocutaneous anastomosis but patients with associated HD continue to suffer from constipation despite adequate reconstructed anorectal canal. They found that most patients in their report had delayed diagnosis of HD as surgeons blamed surgical correction of ARM for the constipation.

Pant et al. [16] collected the clinical, radiological, and surgical findings reported in the literature which may indicate the association of HD in patients with ARM. They found that specific clinical indicators facilitated the early detection of associated HD before complete ARM correction in nine cases. These indicators included colonic narrowing during neonatal colostomy (one case), a nonfunctioning proximal stoma following colostomy (four cases), and caliber changes in the colon observed on distal colostogram (three cases). In contrast, HD was identified after ARM repair in 18 cases, with findings such as a grossly dilated proximal bowel at colostomy closure (one case), persistent abdominal distension, vomiting, recurrent constipation, and enterocolitis (11 cases), the presence of a transition zone on barium enema (two cases), and inadequate dye evacuation following barium enema (five cases). They suggested that identification of the corkscrew colonic vasa recta in cases of high ARM, especially in a segment resembling a transition zone, could prompt the surgeon to at least suspect an associated HD and to have a biopsy from the site. Identification of an associated HD at this stage will help the surgeon to re-plan his surgery.

Watanatittan et al. [8] recommended that the distal loopogram study which is done routinely for cases with high ARM before the definitive repair procedure should be interpreted not only to identify the level of the rectourinary fistula, but also to look for evidence of associated HD. If the study showed a discrepancy with the transitional zone, this would help in selecting an appropriate pull through procedure and the appropriate level of bowel for a pull through. In case 2, when we reassessed the contrast colostogram which was performed before the second phase of ARM correction operation, we realized that the distal blind segment of the colon was narrow with evident discrepancy than the proximal segment which is different than the usual typical appearance with dilatation of the colonic blind end and significant colonic pocket. Unfortunately, these findings were underestimated and were explained as an artifact or angulation of the view.

Management of this rare association represents a challenge necessitating meticulous surgical planning, taking into account the dual pathology. This complexity in management emphasizes the importance of multidisciplinary care involving pediatric surgeons, gastroenterologists, and radiologists [17, 18]. Several techniques for operative HD correction were reported depending on the length of the affected bowel, localization of the disease, and the previous operations for ARM correction. In a systematic review with 90 cases, the surgical technique was reported in 34 patients and consisted of resection of affected bowel with different types of pull-through procedures in 25 patients, sphincter myectomies in two patients with ultrashort segment HD, and seven patients had only one pull-through operation for concurrent HD and ARM correction [19]. This latter category was accounted for the unexpected finding of HD in biopsies taken from a non-functioning stoma [20], biopsies taken at ARM correction [11, 21, 22], or due to surgical techniques that resected the distal colon [23].

The most commonly performed techniques were Soave pull through and Duhamel procedure in 37% and 33% of cases respectively. The other reported procedures included the transanal colorectal pull through procedure (15%), Swenson pull through (7%) and myectomies (7%) [19]. Many authors reported that they preferred the approach of endorectal Soave pull through to avoid the extrarectal plain which was previously used in the operation for ARM correction [19, 24, 25].

As in all cases with ARM, these patients have an absent anus and a malformed sphincter mechanism in addition to the associated aganglionic rectosigmoid segment of variable length. Based on this fact, some authors believed that excision of the aganglionic segment and bringing down the proximal normal ganglionic bowel as a neoanus in these patients would be equivalent to a perineal colostomy. Thus, they suggested preserving the distal bowel (rectum) as a reservoir is essential to improve the future continence. They performed a retrorectal (Duhamel) procedure preserving the previously pulled-through aganglionic segment. This technique involves minimal burden for the already compromised anal sphincter and bladder innervations [16, 19]. In a single case report, the transitional zone was pulled down as the neoanus. The authors reported that with a follow-up period of 4 years, the patient achieved normal bowel movements consisting of 3 to 5 well-formed stools per day with steady growth at the third percentile [21]. The authors stated that although this approach appears risky, it seems to be justified by the result.

According to the same concept, in our technique, we preserved the distal 3–4 cm of the neoanal canal which is known to be aganglionic aiming to compensate for the compromised sphincteric mechanism. This concept helped to achieve a reasonable level of continence in our 3 cases. However, these data are very insufficient to make a generalized statement or a meaningful comparison from the available literature due to the small number of cases and the very low worldwide incidence. A further advantage of this technique is the preservation of the previously constructed neoanus, thereby avoiding excision that could lead to disruption and fibrosis of the adjacent pelvic floor muscles. This approach minimizes the risk of compromising the potential for future continence.

Conversely, in Case 4, the diagnosis of HD was established prior to the planned PSARP procedure. Given this early identification, it was deemed clinically appropriate to perform the pull-through at the level of the ganglionic colon. This approach ensured that only a well-innervated, functionally intact segment of the colon was used for reconstruction, thereby optimizing postoperative bowel function and reducing the risk of complications associated with residual aganglionic segments.

The postoperative outcome regarding continence after ARM and HD correction was surprisingly better than in patients with ARM alone. In a review of 90 cases, Hofmann and Puri [19] found that 88% of the patients followed up were continent after at the end of the follow-up period. However, these good results may be explained as 69% of these patients had a low ARM. Eltayeb et al. [25] reported in their series of 11 cases, that 72% of patients showed continence scores were fair or good. However, the limitation of this study was that the mean follow-up period was only 36 months. In our cases, we observed similar positive outcomes, with all three patients achieving good continence following the modified Swenson-like pull-through procedure. Notably, even the patients with a high ARM (Cases 1 and 2) demonstrated satisfactory bowel control, reporting no soiling episodes and regular bowel movements. These favorable results may be attributed to the individualized bowel management programs implemented postoperatively, as well as the preservation of distal aganglionic segments to maintain continence. However, due to the relatively short follow-up period, large number of patients are needed to fully assess the sustainability of these outcomes, particularly in terms of continence and quality of life as the patients age.

Although this case series provides valuable insights into the rare association of ARM and HD, it is important to acknowledge several limitations. First, the small sample size of only three cases limits the generalizability of our findings. Given the rarity of the ARM-HD association, larger multicenter studies are needed to validate our surgical approach and outcomes. Second, the follow-up periods vary across cases, and while they provide an encouraging outlook, longer-term follow-up is necessary to assess sustained outcomes such as bowel function, continence, and quality of life into adolescence and adulthood. Additionally, our findings may be influenced by regional healthcare resources and patient characteristics, potentially limiting the applicability of these results to broader or more diverse populations. Despite these limitations, this case series contributes to the existing literature by providing detailed diagnostic and surgical strategies for managing this rare association, offering a foundation for future studies.

## Conclusions

The coexistence of ARM and HD is a rare but clinically significant association that presents unique diagnostic and therapeutic challenges. This case series highlights the importance of maintaining a high index of suspicion for HD in patients with ARM, particularly in those with persistent postoperative symptoms such as constipation, abdominal distension, or recurrent enterocolitis. Early recognition through contrast studies and timely rectal biopsies can facilitate prompt diagnosis and intervention, potentially reducing morbidity and improving outcomes.

The Swenson-like pull-through procedure, as described in this series, offers a viable surgical approach for managing this rare association, particularly in cases where HD is diagnosed after ARM repair. By preserving the distal aganglionic segment of the neoanal canal, this technique aims to optimize continence and minimize disruption to the pelvic floor muscles. The favorable postoperative outcomes observed in our cases, including good bowel control and minimal soiling, underscore the potential benefits of this approach.

## Data Availability

The datasets generated during and/or analyzed during this study are available from the corresponding author on reasonable request.

## Abbreviations

ARM: Anorectal malformations
ASD: Atrial septal defect
HD: Hirschsprung's disease
PICU: Pediatric intensive care unit
PSARP: Posterior sagittal anorectoplasty
